# *Vitis vinifera* L. varieties (cv. Cabernet Sauvignon and Chardonnay) vary in leaf water flux in response to elevated CO_2_ growing conditions and a gradual water deficit

**DOI:** 10.1093/aobpla/plaf011

**Published:** 2025-03-08

**Authors:** Alessandra Zuniga, Amélie C M Gaudin, Matthew E Gilbert, Molly E Clemens, Donatella Zona, Walter C Oechel

**Affiliations:** Global Change Research Group, Department of Biology, San Diego State University, 5500 Campanile Drive, San Diego, CA 92182, USA; Department of Plant Sciences, University of California, Davis, 1 Shields Avenue, Davis, CA 95616, USA; NOAA EPP/MSI Center for Earth System Sciences and Remote Sensing Technology, City University of New York, 160 Convent Avenue, New York, NY 10031, USA; Department of Plant Sciences, University of California, Davis, 1 Shields Avenue, Davis, CA 95616, USA; Department of Plant Sciences, University of California, Davis, 1 Shields Avenue, Davis, CA 95616, USA; Global Change Research Group, Department of Biology, San Diego State University, 5500 Campanile Drive, San Diego, CA 92182, USA; Global Change Research Group, Department of Biology, San Diego State University, 5500 Campanile Drive, San Diego, CA 92182, USA; Global Change Research Group, Department of Biology, San Diego State University, 5500 Campanile Drive, San Diego, CA 92182, USA

**Keywords:** water use efficiency, grapevine, elevated CO2, water deficit, evapotranspiration

## Abstract

Grapevine (*Vitis vinifera* L.) stomata are highly sensitive to atmospheric changes and influence the tradeoff between water and carbon, as estimated by intrinsic water use efficiency (*i*WUE). The aim of this study was to examine how elevated CO_2_ concentrations and water deficit affect the *i*WUE and whole plant evapotranspiration of two grapevine varieties (cv. Cabernet Sauvignon and cv. Chardonnay). Dormant cuttings were collected from a vineyard in Temecula Valley, CA, and were grown in a growth chamber under one of two CO_2_ treatments: near ambient (410 ppm) or elevated (700 ppm). After 8 weeks of vegetative growth, grapevines were subjected to a well-watered (25% soil water content [SWC]) or gradual water-deficit treatment implemented over 12 days. We measured leaf gas exchange, including photosynthesis (*A*_net_), stomatal conductance (*g*_s_), intercellular carbon (C_*i*_), and calculated *i*WUE (*A*_net_/*g*_s_), as well as daily cumulative evapotranspiration per unit leaf area (g cm^−2^ day^−1^). Vines were harvested to determine total dry weight, root mass fraction, and nitrogen content. We found that elevated CO_2_ and water deficit interactively increased the *i*WUE for both varieties, with Cabernet Sauvignon having 20% greater *i*WUE than Chardonnay at ~5% SWC. Chardonnay exhibited greater maximum conductance, and 43% more water transpired than Cabernet Sauvignon under a well-watered treatment. Chardonnay plants were also more impacted by elevated CO_2_ and water-deficit treatment than Cabernet Sauvignon, exhibiting greater stomatal sensitivity under these treatments. At ambient CO_2_, water deficit negatively impacted Chardonnay’s photosynthesis than Cabernet Sauvignon. However, this effect was not observed at elevated CO_2_. This study elucidates the intraspecific differences in stomatal behaviour, productivity, and water use of two *V. vinifera* L. genotypes (Cabernet Sauvignon and Chardonnay), under elevated CO_2_ concentrations and short-term water deficit.

## Teaser

This study examines how increasing atmospheric CO_2_ levels and limited water availability affect water use efficiency and overall water loss in two popular grapevine varieties: Cabernet Sauvignon and Chardonnay. Researchers found that both varieties improved their water use efficiency under high CO_2_ and water-deficit conditions, though Cabernet Sauvignon was more conservative. Chardonnay used more water overall and was more impacted by changes in CO_2_ and water levels than was CabernetSauvignon. The findings reported here highlight the nuances of grapevine stomatal response, suggesting that different varieties respond uniquely to environmental change, which could have implications for vineyard management. Growers may need to choose varieties based on their ability to withstand drought and rising CO_2_ conditions. This research underscores the importance of understanding plant responses to climate stress, as it could inform strategies for sustainable viticulture in regions with increasingly limited water supply.

## Introduction

Atmospheric carbon dioxide (CO_2_) concentrations have reached a historical high recording 427 ppm in May of 2024 according to the NOAA Global Monitoring Laboratory (https://gml.noaa.gov/ccgg/trends/). Unless drastic policy measures are taken to reduce anthropogenic footprints, CO_2_ concentrations in the mid-troposphere are projected to reach an estimated 700 ppm by the end of this century (Collins *et al.* 2013). The consequent radiative forcing of elevated atmospheric CO_2_ and other long-lived greenhouse gases will drive the continued rise of average global surface temperature and changes in precipitation patterns. With the expected 2°C rise in global temperature, arid and semi-arid regions will experience increased heat and water stresses (IPCC 2023). These changes are already well underway, evidenced by an increasingly limited water supply and slowed growth of agricultural production in regions at mid to low latitudes in the past 50 years (IPCC 2023). These climate challenges may drastically modify how perennial crops such as grapevines (*Vitis vinifera* L.) access and utilize water for growth.

Stomatal behaviour has previously been described as a dichotomy of hydric behaviours (Tardieu and Simmoneau 1998; Schultz 2003). Isohydric plants maintain a narrow range of leaf water potential, limiting water loss through the early reduction of stomatal conductance, and anisohydric plants exhibit persistently high stomatal conductance when well-watered and under moderate stress conditions (Schultz 2003), and a greater range in leaf water potential (Sade *et al.* 2012). While grapevine varieties are often classified as either isohydric or anisohydric (Hugalde and Vila 2014; Gutiérrez-Gamboa *et al*. 2019), others have suggested they may fall somewhere along this continuum (Klein 2014). More recent theory suggests hydraulic behaviour does not strictly conform to one of these two extremes but rather is an outcome of the environment (Hochberg *et al.* 2018), level of drought stress (Tamayo *et al.* 2023), and the metric used to describe the behaviour (Serrano *et al.* 2024). Despite some studies cross-comparing how elevated CO_2_ and water deficit interact to influence the physiological and yield outcomes of grapevine varieties (Kizildeniz *et al.* 2015, 2018, 2021), largely absent from the discussion is how CO_2_ concentration and water-deficit stress interact to modulate hydric behaviour as well as subspecific differences. This is an important trait to understand in the context of climate adaptations.

At the leaf level, stomatal conductance (*g*_s_) governs the rate at which CO_2_ and H_2_O are exchanged between the atmosphere and inner leaf spaces. We define the cost–benefit ratio as the intrinsic water use efficiency (*i*WUE), calculated as the units of carbon fixed per unit of water transpired via the stomata (Briggs and Shantz 1913; Hatfield and Dold 2019). Under ambient CO_2_ concentrations, reducing stomatal conductance in response to low water potentials may improve *i*WUE in the short term (Bacon 2009). However, lowered conductance simultaneously restricts the diffusion of CO_2_ inside the leaf, limiting photosynthesis and lowering *i*WUE (Bacon 2009), with potential consequences for growth and productivity (Farquhar and Sharkey 1982). For C3 crops, *i*WUE and productivity generally tend to increase when grown in an elevated CO_2_ environment (Policy *et al.* 1993; Poorter *et al.* 2022) due to the carboxylating enzyme ribulose-1,5-bisphosphate carboxylase/oxygenase (Rubisco) being limited at present CO_2_ concentrations (Long and Drake 1992). An increase in *i*WUE is partially explained by an increase in the concentration gradient of CO_2_ between internal leaf spaces and the atmosphere, which increases the rate at which CO_2_ diffuses into leaf spaces. There is conflicting evidence regarding how elevated CO_2_ and water deficit should interactively impact *i*WUE, indicating it is a mechanism that is largely context dependent (Xu *et al.* 2016).

A higher rate of CO_2_ diffusion affords reduced stomatal conductance (Cowan 1978) and thus lower water loss via transpiration (Poorter *et al.* 2022). A review by Ainsworth and Rogers (2007) reports a ~25% reduction in conductance along with a ~18% increase in yield observed for C3 crops grown under elevated CO_2_ conditions when no known stressors were present. In a montane grassland Free Air CO_2_ Enrichment (FACE) system, elevated CO_2_ increased stomatal resistance (~50%) with a consequent decrease in daily evapotranspiration (~10%) associated with stomatal closure, allowing drought mitigation (Vremec *et al.* 2023). Similarly, maize crop grown in an elevated CO_2_ environment have shown a 9% reduction in evapotranspiration (Hussain *et al.* 2013) and an increase in soil moisture (Leakey *et al.* 2006).

Studies examining grapevine response to an elevated CO_2_ environment demonstrate an increase in primary productivity (Bindi *et al.* 2001), development of fine roots (Wohlfahrt *et al.* 2018), and fruit yield (Bindi *et al.* 1996; Wohlfahrt *et al.* 2018), as well as changes to berry chemistry (Arrizabalaga-Arriazu *et al.* 2021). At the leaf level, there is a significant downregulation of carbon assimilation (Salazar-Parra *et al*. 2012, 2015; Kizildeniz *et al.* 2021), likely attributed to leaf nitrogen dilution (Leibar *et al.* 2015; da Silva *et al.* 2017; Kizildeniz *et al.* 2021). When combined with other stressors such as heat, elevated CO_2_ is shown to reduce whole-season transpiration of grapevines, compensating for the increased transpiration under warmer temperatures (Edwards *et al.* 2016). In an open-top chamber, elevated CO_2_ and increased temperature did not affect conductance but significantly increased photosynthetic rates in grapevines (Edwards *et al.* 2017).

Current understanding of the impacts of atmospheric CO_2_ on grapevine water balance and productivity largely derives from FACE experiments, particularly on Cabernet Sauvignon and Riesling varieties (Wohlfahrt *et al.* 2018, 2022), and greenhouse studies on red and white Tempranillo (Kizildeniz *et al.* 2015, 2018, 2021). As of now, findings are inconsistent with some studies suggesting whole-plant WUE of grapevines decreases (Douthe *et al.* 2018), while others merely found a transient increase in *i*WUE (da Silva *et al.* 2017; Wohlfahrt *et al.* 2018) in response to drought and high CO_2_ treatments. Field studies are often confounded by soil variability (Groß *et al.* 2023), steep microclimate gradients, as well as biotic pressures, while open-top chambers and greenhouse studies are posed with microclimate effects (Moutinho-Pereira *et al.* 2010). Examining how an elevated CO_2_ environment and water deficit simultaneously impact the *i*WUE of grapevines requires precision control of the many factors that influence stomatal regulation, including light levels, temperature, soil moisture, and humidity.

Here, we aim to understand the independent and interactive effects of elevated CO_2_ and water deficit on stomatal behaviour and whole vine water use of two grapevine varieties grown in a walk-in chamber, where we controlled CO_2_, temperature, light, and humidity. We used cv. Cabernet Sauvignon and cv. Chardonnay, with known isohydric (Hochberg *et al.* 2013; Tramontini *et al.* 2014) and anisohydric (Vandeleur *et al.* 2009; Pou *et al.* 2012; Gutiérrez-Gamboa *et al.* 2019) hydric strategies, respectively. Our research questions were:

How do elevated CO_2_ and gradual water deficit interact to increase the *i*WUE in vines? To what extent do stomatal conductance and photosynthesis regulate this ratio? Which of the two *V. vinifera* L. varieties has the most conservative leaf level and whole-plant level water usage under these treatments*?*

The cultural distribution of grapevine varieties across the globe has resulted in a vast range of stomatal response mechanisms (Prieto *et al.* 2010; Bota *et al.* 2016). As the suitability of current wine-growing regions is shifting (Monteverde and de Sales 2020; van Leeuwen *et al.* 2024), this phenotypic diversity can be harnessed for climate adaptation purposes (Morales-Castilla *et al.* 2020). In this study, we determine nuanced differences in the water use strategies between Cabernet Sauvignon and Chardonnay, two *V. vinifera* L. varieties commonly grown in semi-arid regions. Identifying variability in stomatal responses and how it affects WUE may guide grape growers in selecting more drought-tolerant varieties adapted to future climate conditions. This information may also guide genetic engineering and selective breeding programs supporting the sustainability and resilience of viticulture to climate change.

## Materials and methods

**Figure 1.**
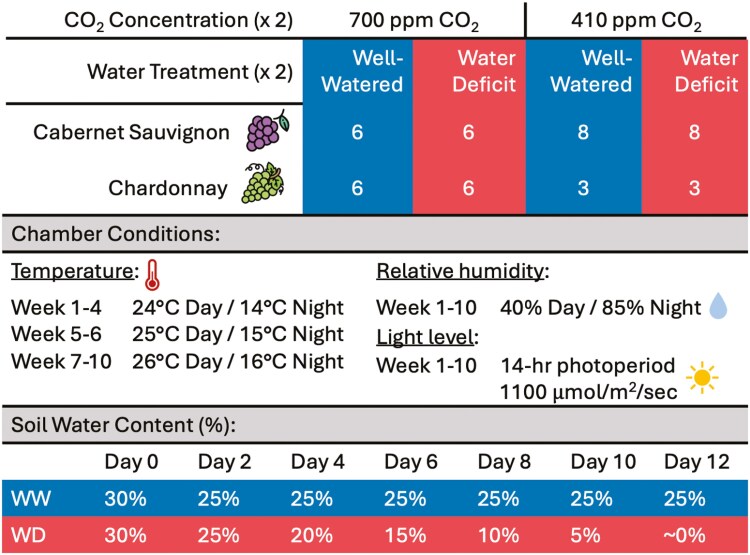
Experimental design testing effects of two CO_2_ concentrations (700 and 400 ppm) and two water treatments (well-watered and water deficit), on two grapevine varieties (Cabernet Sauvignon and Chardonnay). Growth chamber conditions, including temperature (°C), relative humidity (%), and light levels, were controlled and set to mimic day/night cycles simulating the spring climate of the Temecula region, CA, USA. Soil water content (%) was maintained at 25% for the well-watered (WW) treatment and was gradually reduced gravimetrically for the water-deficit (WD) treatment over 12 days, as shown in the bottom table.

The experiment was arranged as a three-factor factorial design with two CO_2_ concentrations (700 and 410 ppm), two water treatments (well-watered control and water-deficit), and two grapevine varieties (cv. Cabernet Sauvignon and cv. Chardonnay). A summary of treatments and the number of replicates per treatment are shown in Fig. 1.

### Plant material

We collected dormant cuttings of Cabernet Sauvignon and Chardonnay from Wilson Creek Winery in Temecula, CA, USA (33.5472° N, 117.0448° W) in January of 2021. Cuttings were selected from previous 1-year-old wood on the scion that was approximately 2 cm in diameter and 30–40 cm long with six nodes on each. Sixty cuttings of each variety were sampled and placed in a container with moist mulch chips and kept in dark, cold storage at 4.5°C to continue dormancy (Anzanello *et al.* 2018).

### Growth chamber experiment establishment

Plants were grown in a walk-in growth chamber (BDW40, Conviron Plant Growth Chamber, Manitoba, Canada) at San Diego State University. Top-down airflow and spectral aluminium-lined walls in the chamber allow for an even distribution of airflow and light through the plant canopy. Due to the chamber having only one growing area in which the CO_2_ concentrations could be manipulated, the study was divided into two asynchronous CO_2_ cohort treatments. In March 2020, half of the dormant grapevine cuttings in storage were randomly selected for the first experiment under elevated CO_2_ conditions (700 ppm). The remaining cuttings were kept in cold storage at 4.5°C to continue dormancy and rooting was induced 2 months later for the second experiment where grapevines were grown at near ambient CO_2_ conditions (410 ppm).

Cuttings were dipped into indole-3-butyric acid (0.10%) and were placed vertically on a mulch bed and incubated at 30°C in a dark room to induce root development. After 30 days in incubation, the bottom tips of cuttings were checked for callus from which roots started to emerge. When rooting was observed on 80% of cuttings, 20 cuttings of Cabernet Sauvignon and Chardonnay with visible rooting success were selected and transferred to the growth chamber for 70 days (10 weeks) under each CO_2_ treatment (700 and 410 ppm). All other environmental variables in the chamber were controlled and were selected to mimic day/night cycles and simulate the spring climate conditions of the Temecula region, CA, USA (Fig. 1).

Cuttings were planted in 2.5 L pots with approximately 1.2 kg of a prepared soil medium made of commercial potting soil (Vigoro All Purpose Potting Mix), sand, and coarse grade perlite in a 1:2:1 ratio. The purpose was to allow for both good drainage and the retention of soil moisture levels to control soil water content (SWC) levels. After saturating the soil and allowing it to percolate for 30 minutes from pots, it was determined that the SWC was 30% at field capacity. Cuttings were planted vertically in pots with three nodes beneath the soil surface and the remaining three nodes above the soil. Pots were randomly arranged and shuffled inside the chamber twice a week to eliminate potential edge effects.

All pots were maintained well-watered for the first 56 days after planting by saturating the soil every 2–3 days at the same time of day. Vines were monitored daily for phenological progression, and we recorded the timing of bud break, shoot, and leaf development that occurred on each replicate. Vines produced two to three shoots from the nodes, and number coding was used to indicate the stage of phenology according to Coombe (1995) as follows: (0) dormancy, (1) early bud swell, (2) late bud swell, (3) bud burst, (4) first visible leaves, (5) 2–4 cm shoots, and (6) 10–20 cm shoots and fully expanded leaves, after which vines experienced tapered growth. Successful vines that reached the sixth stage of phenological development after 56 days were selected for the experiment. For unknown reasons, the propagation success of the two varieties differed under ambient CO_2_ conditions, and thus, fewer replicate plants for Chardonnay proceeded for the water treatments than Cabernet Sauvignon, resulting in an unbalanced design.

### Water treatments

On day 56 before initiating water treatments, all pots were irrigated to full saturation, allowed to percolate for 30 minutes, and then weighed to determine individual pot weight (g) at field capacity. This is consequently referred to as day 0 of the water treatments. Pots were weighed daily, and the change in the pot weight was attributed to water loss via evapotranspiration (Duan *et al.* 2014). Water treatments were controlled gravimetrically daily by replacing the amount of water lost (g) based on initial weight at field capacity and converted to SWC (%), where 30% SWC is equivalent to field capacity. On day 2, both water treatments were dropped to 25% SWC, after which well-watered pots were maintained at 25% SWC by rewatering the amount of daily water loss for the remainder of the experiment. This ensured well-watered plants had regular access to water while avoiding the potential effects of waterlogging if kept at 30% SWC. Water-deficit treated pots were allowed to dry down naturally due to evapotranspiration and were only rewatered to achieve a 5% decrease in SWC every other day until reaching near 0% on day 12, after which watering was suspended for 2 days before harvesting (Fig. 1). The average accumulated water applied per treatment throughout the water-deficit experiment was also analysed and is displayed in Table 1.

**Table 1.** Average accumulated water added per treatment throughout a 12-day gradual water-deficit experiment

CO_2_ conc. (ppm)	Water treatment	Variety	Sample size (*n*)	Mean (g of water)	SE
700	well-watered	Cabernet Sauvignon	6	867.26	28.02
		Chardonnay	6	1024.81	86.90
	water deficit	Cabernet Sauvignon	6	171.91	23.80
		Chardonnay	6	163.70	31.92
400	well-watered	Cabernet Sauvignon	8	1092.31	39.78
		Chardonnay	3	1046.29	41.43
	water deficit	Cabernet Sauvignon	8	213.18	15.24
		Chardonnay	3	205.28	90.86

Well-watered vines were maintained at a 25% SWC, and water-deficit vines were gradually reduced to near ~0% SWC by day 12.

### Leaf gas exchange

Leaf gas exchange metrics including net assimilation (*A*_net_), stomatal conductance (*g*_s_), and intercellular carbon (C_*i*_) values were collected using a Li-6400 (LiCOR Sci. Inc., Lincoln, NE, USA). The leaf cuvette was set to flow rate = 400 μmol s^−1^, temperature = 26°C, PAR = 1100 μmol m^−2^ s^−1^, area = 6 cm^2^, stomatal ratio = 0, and reference CO_2_ = 410 and 700 ppm, for the ambient and elevated CO_2_ treatments, respectively. Two leaves, one shade and one sun leaf, were selected from each replicate vine for measurements. Measurements were taken on day 0 of water treatments when all vines had a SWC of 30% and subsequently, when deficit treatment reached 25%, 15%, 5%, and near 0% SWC. All measurements were taken in the morning after 4 hours of exposure to light and 1 hour after pots were watered. *i*WUE was calculated at each time point by taking the ratio between net carbon assimilation (*A*_net_) and water loss through transpiration as observed through stomatal conductance (*g*_s_) (Briggs and Shantz 1913). We chose to analyse *i*WUE (*A*_net_/*g*_s_) over instantaneous WUE (E/*A*_net_) because *g*_s_ is more accurate for evaluating variation among crop genotypes, while E is compromised by environmental variability (Leakey *et al.* 2019). Day 10 of the water treatments was analysed as a point measurement, at which water-deficit-treated plants had reached near 5% SWC. Due to the unreliability of photosynthesis measurements at low stomatal conductance (Hussain *et al.* 2024), day 12 measurements, when SWC reached ~0%, were removed from the analysis of leaf-level gas exchange.

### Evapotranspiration metrics

Daily pot evapotranspiration was determined by weighing pots gravimetrically at the same time every day to determine water loss as grams of water per 24-hour period (g day^−1^) at each stage of the watering treatments. To standardize the average amount of water transpired, the total leaf area (cm^2^) of the plant was divided by the grams of water loss over a 24-hour period (g day^−1^) to determine daily cumulative evapotranspiration per unit leaf area (g cm^−2^ day^−1^).

### Leaf area, biomass, and elemental composition

After each 10-week experiment, mature plants were harvested, and divided by roots, green biomass, and wood. Total leaf area (cm^2^) was analysed once after the watering treatments on Image J using top-down pictures of all harvested leaves flat onto a white paper background with a 58.06 cm^2^ calibration ruler. Roots and vegetative biomass were oven-dried at 80°C until stable dry weight. Root and shoot material were used to determine total dry weight as well as root mass fraction. Dried leaf tissue was packed into tin capsules to determine the total carbon and nitrogen (g N g^−1^ leaf) by mass spectrometry using a PDZ Europa ANCA-GSL elemental analyser interfaced to a PDZ Europa 20-20 isotope ratio mass spectrometer (Sercon Ltd., Cheshire, UK) at the UC Davis Stable Isotope Facility.

### Statistical analysis

Statistical analysis and data visualization were completed using R statistical software version 4.0.4 and R studio version 2023.06.1 + 524 (R Development Core Team, Vienna, Austria). Leaf response variables (*i*WUE, *g*_s_, *A*_net_, C_*i*_) were collected at four time points as the water deficit was gradually imposed. We used a linear mixed effects model using the function ‘lme’ in the *nlme* package to test for the effects of each fixed factor (CO_2_, water treatment, grapevine variety) and their interaction on leaf responses (*i*WUE, *g*_s_, *A*_net_, C_*i*_). Due to repeated measures and gradual implementation of water-deficit treatment, plant ID was added as a random factor. A log transformation was used in cases where the data exhibited a positive skew to the right, bringing distribution closer to normality. A linear model using the ‘lm’ function was used to analyse the effects of fixed factors on leaf area, total biomass, evapotranspiration, and leaf elemental composition (C:N) at harvest. Significance was tested using a type III, three-way ANOVA to test for the interactive and fixed effects of CO_2_, water treatment, and grapevine variety on all leaf and vine response variables while accounting for an unbalanced dataset. Results were considered statistically significant at *P* <.05. Means differences were calculated post-hoc using the *emmeans* package when effects were significant in the full model. All data are displayed as means ± SE. A regression model was used to determine the correlation between stomatal conductance (*g*_s_) and evapotranspiration for each grapevine variety. The relationship was best represented by a polynomial regression, where the strength of the relationship between the two responses was explained using an *R*^2^ and *P*-value <.05. It is important to note that this study included a relatively small sample size, which may limit the generalizability of the findings.

## Results

### Leaf gas exchange responses

**Figure 2.**
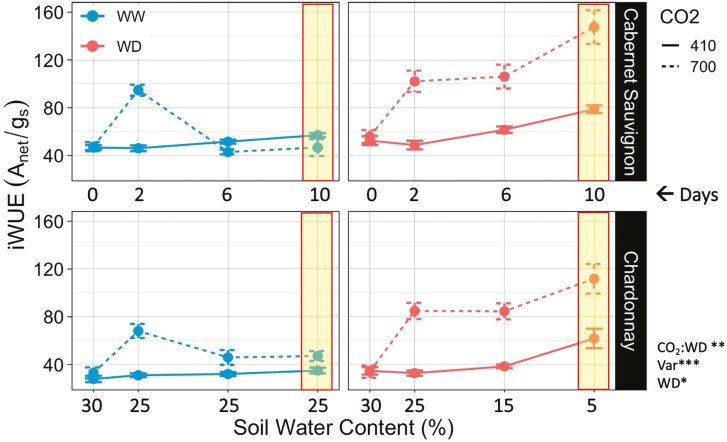
Intrinsic water use efficiency (*i*WUE) measured as a ratio between carbon assimilation (*A*_net_) and stomatal conductance (*g*_s_) of Cabernet Sauvignon (CS) and Chardonnay (CH) grapevine varieties grown in near ambient (410 ppm) and elevated (700 ppm) CO_2_ conditions and exposed to well-watered (WW) and water-deficit (WD) treatments across 10 days. Gravimetric soil water content was decreased to 5% SWC for the WD treatment and maintained at 25% SWC for the WW treatment. Points on the graph represent means ± SE (700 ppm: *n* = 6; 410 ppm × CS: *n* = 8; 410 ppm × CH: *n* = 3) of measurements taken on days 0, 2, 6, and 10 of the water treatments. Day 10 of deficit treatment (near 5% SWC) resulted in the greatest interactive effect. Asterisks indicate significant treatment effects (alpha = .05; ***P < .001; **P < .01; *P < .05).

A gradual water deficit increased the *i*WUE for both varieties under both CO_2_ concentrations (*P* < .05). However, the interaction between elevated CO_2_ and water-deficit treatments resulted in the greatest increase in *i*WUE for both Cabernet Sauvignon and Chardonnay vines (*P* < .01) (Fig. 2). The largest effect size was on the 10th day of the watering treatments (indicated by yellow shading), when water-deficit soils reached near 5% SWC.

**Figure 3.**
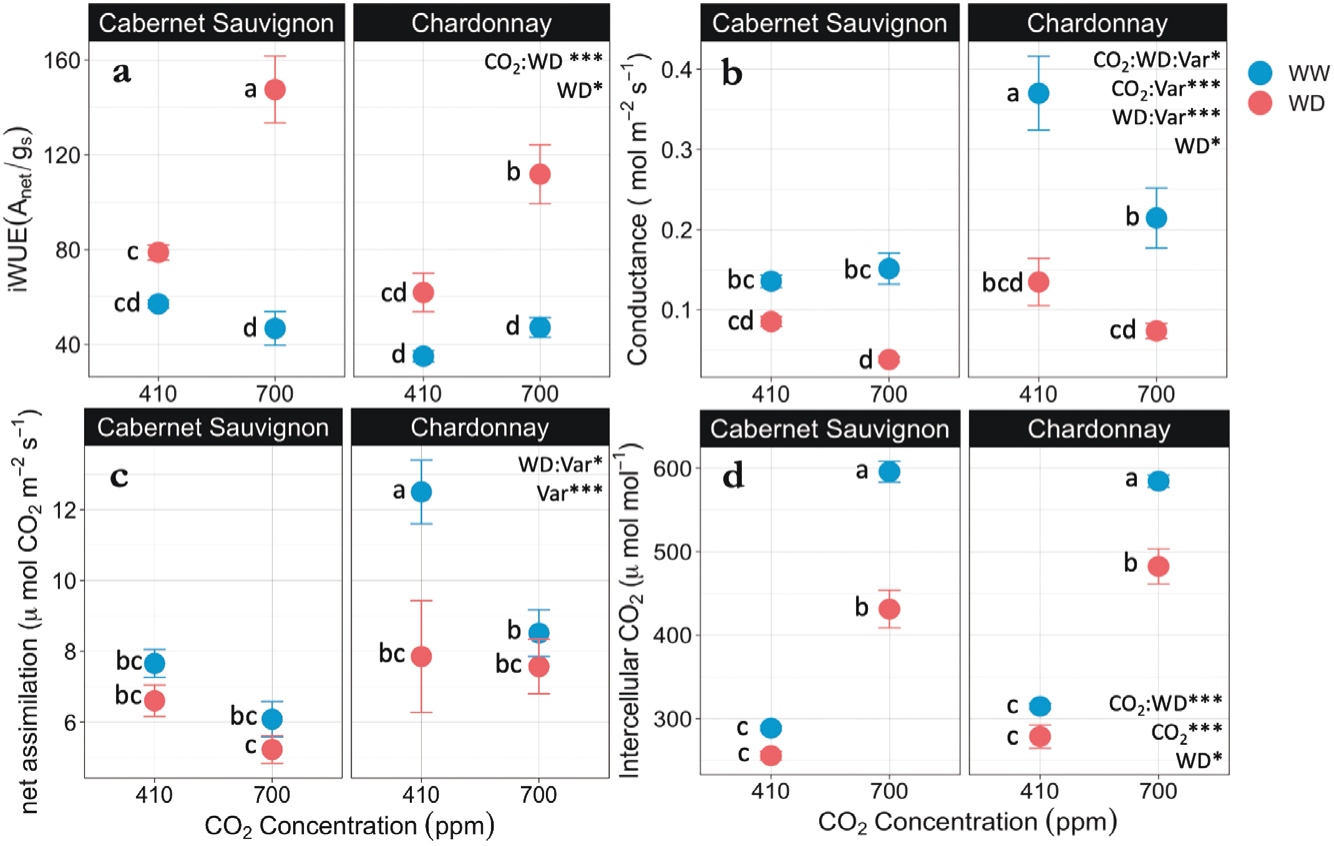
(a) Intrinsic water use efficiency (*i*WUE), (b) leaf stomatal conductance, (c) net assimilation, and (d) leaf intercellular carbon (C_*i*_) of Cabernet Sauvignon (CS) and Chardonnay (CH) grapevine varieties (Var) grown under ambient (410 ppm) and elevated (700 ppm) CO_2_ conditions and exposed to well-watered (WW) and water-deficit (WD) treatments after 10 days. Points on the graph represent treatment means ± SE. For all panels, 700 ppm: *n* = 6; 410 ppm × CS: *n* = 8; 410 ppm × CH: *n* = 3. Different letters indicate significant differences between the treatment means (alpha = .05). Asterisks indicate significant treatment effects (***P < .001; *P < .05).

On day 10 of the watering treatments, water deficit (5% SWC) increased the *i*WUE for both varieties under both elevated and ambient CO_2_ conditions (*P* < .01) (Fig. 3a). However, the combined effects of elevated CO_2_ and water deficit resulted in significantly greater *i*WUE for both varieties (*P* < .001) although this effect was 20% greater for Cabernet Sauvignon than the Chardonnay (*P* = .05).

On day 10 of the watering treatments, the effects of elevated CO_2_ and water deficit on leaf stomatal conductance were variety specific (*P* < .05) (Fig. 3b). Water deficit significantly reduced stomatal conductance (*P* < .05), and this negative effect was greater for Chardonnay versus Cabernet Sauvignon under the ambient CO_2_ conditions (*P* < .001). The stomatal conductance of Chardonnay vines was lower under elevated CO_2_ conditions versus ambient, particularly in the well-watered treatment (*P* < .001), while this effect was not observed for Cabernet Sauvignon. Stomatal conductance was 148% greater for Chardonnay than Cabernet Sauvignon under ambient, well-watered conditions (*P* < .001). These differences between varieties were not observed under the elevated CO_2_ treatment.

On day 10 of the watering treatments, the effect of water deficit on net assimilation was variety specific (*P* < .05). Water deficit had a significant negative effect on assimilation for Chardonnay plants under ambient CO_2_ conditions (*P* < .001), but this effect was not observed at elevated CO_2_. Water deficit did not have a significant negative effect on the assimilation rates of Cabernet Sauvignon vines in either ambient or elevated CO_2_ conditions (Fig. 3c). Net assimilation was downregulated by 32% in well-watered Chardonnay vines grown under an elevated CO_2_ environment in comparison to vines grown under ambient CO_2_ conditions (*P* < .001), while this was not significant for Cabernet Sauvignon.

Leaf intercellular CO_2_ concentrations (C_*i*_) were significantly greater for vines grown in an elevated CO_2_ environment in comparison to ambient CO_2_ (*P* < .001). On day 10 of the watering treatments, water deficit reduced the intercellular CO_2_ (C_*i*_) for both varieties (*P* < .05); however, this effect was compensated by 63% and 57% for Cabernet Sauvignon and Chardonnay when grown under elevated CO_2_ conditions, respectively (*P* < .001 and *P* < .001) (Fig. 3d).

### Evapotranspiration per unit leaf area

**Figure 4.**
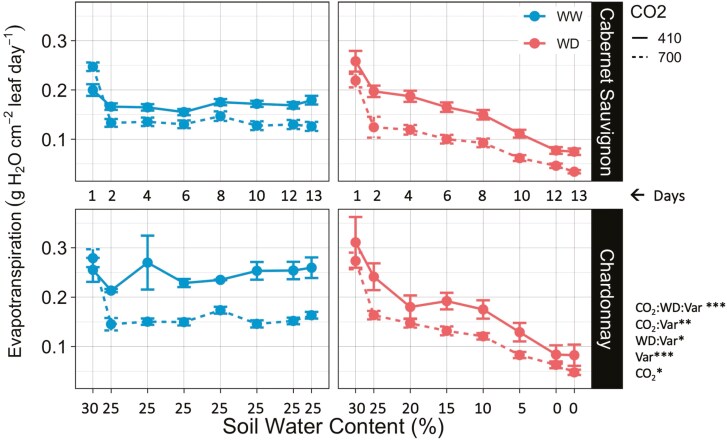
Daily cumulative evapotranspiration per unit leaf area averaged for Cabernet Sauvignon and Chardonnay grapevines grown under ambient (410 ppm) and elevated (700 ppm) CO_2_ conditions and exposed to well-watered (WW) and water-deficit (WD) treatments across 12 days. Gravimetric soil water content (%) is represented on the x-axis. Points on the graph represent treatment means ± SE (700 ppm: *n* = 6; 410 ppm × CS: *n* = 8; 410 ppm × CH: *n* = 3). Asterisks indicate significant treatment effects (alpha = .05; ***P < .001; **P < .01; *P < .05).

Daily cumulative evapotranspiration per unit leaf area gradually declined as the water-deficit treatment progressed from 30% SWC to near 0% (Fig. 4). Average total accumulated water added per treatment group throughout the water-deficit experiment can be found in Table 1. Vines grown under elevated CO_2_ conditions had lower evapotranspiration rates under both well-watered and water-deficit treatments compared to ambient CO_2_ conditions (*P* < .05).

A strong interaction was observed between elevated CO_2_ and water deficit on evapotranspiration per unit leaf area, and the effect was different for the two varieties (*P* < .001) (Fig. 4). Under ambient CO_2_ and well-watered conditions, Chardonnay used 43% more water per leaf area per day than Cabernet Sauvignon (*P* < .001). Under ambient CO_2_ conditions, Chardonnay exhibited a significant reduction in transpiration rates per leaf area when exposed to the water-deficit treatment (*P* < .05), while this effect was not significant for Cabernet Sauvignon. The effects of elevated CO_2_ on well-watered Chardonnay were greater than those observed for Cabernet Sauvignon under the same treatment (*P* < .01).

**Figure 5.**
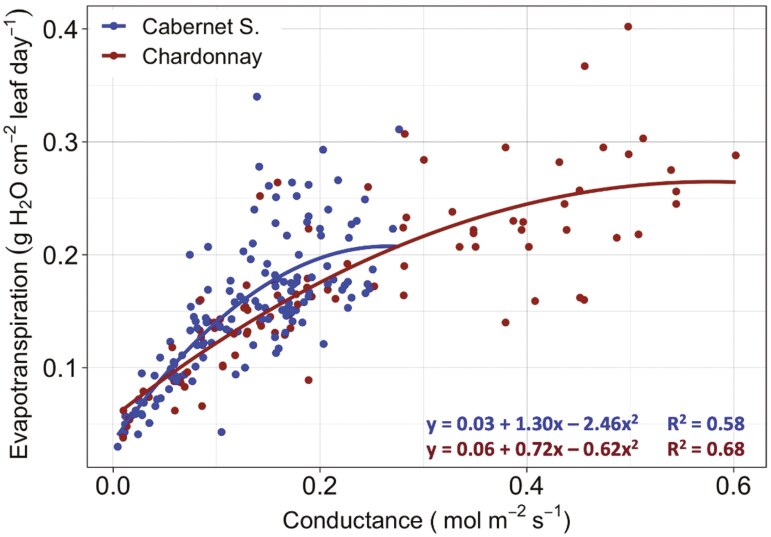
Quadratic relationship between leaf stomatal conductance (*g*_s_) and evapotranspiration per unit leaf area of Cabernet Sauvignon and Chardonnay grapevines. Points on the graph represent conductance measurements and evapotranspiration measurements taken on the same day and the regression lines represent the best fit to the data points with the *R*^2^ values represented on the bottom-right indicating goodness of fit (alpha = .05).

Leaf stomatal conductance (*g*_s_) exhibited a positive quadratic relationship with 24-hour evapotranspiration per unit leaf area (g H_2_O cm^−2^ leaf day^−1^) and this relationship varied by grapevine variety (*P* < .001). For Cabernet Sauvignon and Chardonnay, 58% and 68% of the variation in water loss as measured by evapotranspiration was explained by conductance, respectively (*R*^2^ = .58, *P* < .001; *R*^2^ = .68, *P* < .001) (Fig. 5). As leaf stomatal conductance increases so did the daily amount of water loss in grams per square centimetre of leaf area, a relationship that was similar for the two varieties Cabernet Sauvignon and Chardonnay. However, Chardonnay exhibited a higher range in stomatal conductance values and reached a greater maximum evapotranspiration than did the Cabernet Sauvignon (*P* < .01).

### Leaf area, biomass, and nitrogen composition

**Figure 6.**
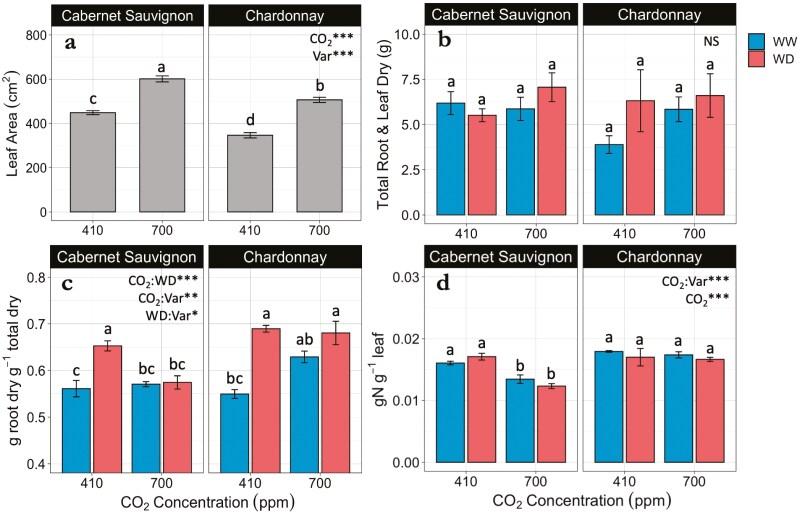
(a) Average total leaf area, (b) total dry mass of roots and leaves, (c) root mass fraction, and (d) total leaf nitrogen. All measurements taken on harvested Cabernet Sauvignon (CS) and Chardonnay (CH) grapevine varieties (Var) grown under ambient (410 ppm) and elevated (700 ppm) CO_2_ conditions and exposed to 12 days of the well-watered (WW) and water-deficit (WD) treatments. Bars represent treatment means ± SE. For all panels 700 ppm: *n* = 6; 410 ppm × CS: *n* = 8; 410 ppm × CH: *n* = 3. Different letters indicate significant differences between the treatment means (alpha = .05). Asterisks indicate significant treatment effects ***P < .001; **P < .01; *P < .05; NS = not significant.

Fixed effects of elevated CO_2_ (*P* < .001) and grapevine variety (*P* < .001) on the total leaf area were significant but not water deficit (Fig. 6a). Cabernet Sauvignon and Chardonnay grown under elevated CO_2_ conditions had 34% and 46% larger leaf areas, respectively, compared to those grown under ambient CO_2_ conditions. Cabernet Sauvignon produced greater total leaf area than Chardonnay under both ambient (*P* < .001) and elevated (*P* < .001) conditions.

No effects were observed for CO_2_ concentration, water treatment, and grapevine variety on the total biomass (Fig. 6b). However, the root mass fraction of vines indicated strong interactive effects between CO_2_ and water treatment (*P* < .001), CO_2_ and variety (*P* < .01), and water treatment and variety (*P* < .05) (Fig. 6c). Vines exposed to water-deficit treatment had greater root mass fraction than vines that were well-watered, except for Cabernet Sauvignon grown at elevated CO_2_. In the elevated CO_2_ treatment, Chardonnay had a greater root mass fraction in both well-watered and water-deficit treatments in comparison to Cabernet Sauvignon (*P* < .01 and *P* < .001, respectively).

CO_2_ concentration and variety had a strong interaction on the total leaf nitrogen (N) concentration (*P* < .001), but water deficit did not. Elevated CO_2_ had a strong negative effect on total leaf N for the Cabernet Sauvignon under both well-watered (*P* < .01) and water-deficit treatments (*P* < .001) (Fig. 6d). However, CO_2_ did not have significant effects for the Chardonnay vines. We extrapolated total N in the leaves per plant by multiplying leaf N (g N g^−1^ leaf) by total leaf dry mass (g) and found no differences in total N uptake across treatments (data not shown).

## Discussion

### Elevated CO_2_ promotes *i*WUE, especially when water is limiting

The increase in leaf *i*WUE when grown under an elevated CO_2_ environment (700 ppm) in this study (Fig. 2) is consistent with another study on grapevines (Cabernet Sauvignon and Riesling) (Wohlfahrt *et al.* 2018), as well as other crops such as sorghum (Conley *et al*. 2001), winter wheat (Tuba *et al.* 1994; Allen *et al.* 2011), and soybean (Li *et al.* 2013). Moreover, we find that elevated CO_2_ during vegetative growth along with a gradual water deficit further increased the *i*WUE of grapevines, with the greatest effect on day 10 when soil presented a very low soil moisture content (~5%) (Fig. 3a). This effect has also been observed in other C3 plants such as barley (Robredo *et al.* 2007), wheat (Cao *et al.* 2022), tomato (Pazzagli *et al.* 2016), and verbena sage (Javaid *et al.* 2022). To our knowledge, only one other study tested and found an interactive response to water deficit and elevated CO_2_ resulting in increased *i*WUE for grapevine (da Silva *et al.* 2017), although that study was done on a different grapevine species (*Vitis labrusca*). Evidence supports a positive interaction between elevated CO_2_ and water deficit on *i*WUE. However, the size of this effect may vary across *V. vinifera* L. varieties, which has implications for water management in vineyards. Our study uniquely shows that the magnitude of this effect differed by variety, where Cabernet Sauvignon exhibited a 20% greater *i*WUE than Chardonnay in response to elevated CO_2_ and water deficit (Fig. 3a). Elevated levels of CO_2_ may have a mitigating effect on grapevine water balance, at least short-term as observed in this 2-week water-deficit experiment. However, CO_2_ and water deficit likely interact with other variables to affect grapevine water use and performance over longer periods. A recent review by Clemens *et al.* (2022) suggests that while rising CO_2_ concentrations are expected to increase *i*WUE transiently at the leaf level, scaling up these effects to the whole plant in the field setting remains challenging due to variability in soil water availability, temperature, as well as the grapevine scion–rootstock interaction.

### Response variables underlying the increase in *i*WUE vary by variety

To better understand what drives changes to *i*WUE, it is critical to quantify the extent to which stomatal conductance (*g*_s_) and net photosynthesis (*A*_net_) independently influence the balance of this ratio and how those responses differ between the two varieties. In this study, the increase in *i*WUE observed after 10 days of water deficit was due to reduced stomatal conductance. In addition, we found that elevated CO_2_ levels mediated the closure of stomata for Chardonnay under well-watered conditions (Fig. 3b), which has been supported by other studies (Engineer *et al.* 2016; Ma and Bai 2021). However, results differ from a FACE study finding that stomatal conductance and transpiration were higher for Cabernet Sauvignon and Riesling grapevines exposed to high CO_2_ for three seasons (Wohlfahrt *et al.* 2018). Despite lowered conductance in response to water deficit and elevated CO_2_, intercellular carbon (C_*i*_) was significantly increased for both Cabernet Sauvignon and Chardonnay vines (Fig. 3d).

Photosynthesis (*A*_net_), as the second variable in the *i*WUE equation, can promote a greater *i*WUE by increasing net photosynthesis. However, we found that net photosynthesis was reduced under elevated CO_2_ concentrations, particularly in the well-watered treatment with a greater effect observed in the Chardonnay vines compared to the Cabernet Sauvignon vines (Fig. 3c). This is opposite to field studies finding that the observed increase in *i*WUE in response to elevated CO_2_ was explained by an increase in net assimilation for Riesling, Cabernet Sauvignon (Wohlfahrt *et al.* 2018), and Touriga Franca varieties (Moutinho-Pereira *et al.* 2009), while conductance was unaffected. In fact, a review of FACE studies by Ainsworth and Rogers (2007) indicates that an elevated CO_2_ environment should stimulate the fertilization effect for C3 plants. Interestingly, we did not observe a fertilization effect despite higher concentrations of intercellular carbon (C_*i*_) for vines grown under elevated CO_2_ (Fig. 3d), indicating other photosynthetic limitations may have been at play. For example, photosynthetic acclimation may occur if plants downregulate RuBisCO activity, or other downstream photosynthetic mechanisms, in response to sustained CO_2_ saturation. Some studies have reported a down regulation of photosynthesis at elevated CO_2_ levels for grapevines (Salazar-Parra *et al*. 2012, 2015; da Silva *et al.* 2017; Kizildeniz *et al.* 2021), which were explained by a nitrogen dilution effect (Leibar *et al.* 2015; da Silva *et al.* 2017; Kizildeniz *et al.* 2021). To this end, our study found that although total N per plant did not differ across treatments (Fig. 6d), the leaf area increased while leaf N content decreased under elevated CO_2_ only for the Cabernet Sauvignon (Fig. 6a), pointing to a nitrogen dilution effect. This agrees with studies on cv. Tempranillo grapevines (Arrizabalaga-Arriazu *et al.* 2021), as well as beans (*Phaseolus vulgaris* L.) (Jifon and Wolfe 2002) showing an increase in C:N ratio along with photosynthetic acclimation when grown under elevated CO_2_. We also recognize that sink limitations may play a part in the observed photosynthetic acclimation (Ruiz-Vera *et al.* 2017). This effect may be especially pronounced in potted plants smaller than 8L, which may restrict root growth (Arp 1991), a known limitation of this study.

We find that Chardonnay’s photosynthesis was more negatively impacted by water deficit under ambient CO_2_ concentrations than Cabernet Sauvignon (Fig. 3c), corresponding to the greater negative impact of water deficit on stomatal conductance for Chardonnay (Fig. 3b). A reduction in photosynthesis in response to water deficit has been demonstrated in alternative *V. vinifera* L. varieties, such as Riesling (Bertamini *et al.* 2006), Tempranillo (Flexas *et al.* 1999), and Cabernet Sauvignon (Cramer *et al.* 2013). However, elevated CO_2_ did not alleviate the negative effects of water deficit as we had predicted. Elevated CO_2_ has been found to attenuate the negative effects of drought on vegetative growth for Tempranillo, when compounded with temperature stress (Kizildeniz *et al.* 2015), as well as for *V. labrusca* by improving leaf carbon balance (da Silva *et al.* 2017). However, in a follow-up study by Kizildeniz *et al.* (2018), elevated CO_2_ did not compensate for the negative effects of water stress on vegetative growth in Tempranillo. Rising CO_2_ concentrations have important implications for how plants may cope with increasing droughts, yet a comprehensive understanding of these impacts on grapevine productivity remains limited.

### Elevated CO_2_ promoted larger leaf area, but biomass was unaffected

In this study, CO_2_ fertilization promoted larger leaf areas in both varieties, but the leaf area was greater for Cabernet Sauvignon under both ambient and elevated CO_2_ conditions (Fig. 6a). Despite an expanded leaf area, no differences in total biomass were detected (Fig. 6b), contrary to our predictions. A study on soybeans also found an increase in leaf area under elevated concentrations, but shoot weight was increased (Li *et al.* 2013). An increase in leaf area under elevated CO_2_ conditions may be explained by cell expansion, caused by increases in cell wall plasticity rather than increased cell division (Pritchard *et al.* 1999), and some evidence indicates these effects are genotype-dependent (Taylor *et al.* 2001). We did observe a greater root mass fraction for vines exposed to water deficit when grown at ambient CO_2_ (Fig. 6c). However, contrary to what we expected, this effect was not enhanced in an elevated CO_2_ environment. Similarly, a study on red and white Tempranillo grapevines found drought increased the proportion of biomass allocated to roots, while CO_2_ effects were smaller and variety dependent (Kizildeniz *et al.* 2021). However, the main effects of elevated CO_2_ have broadly shown to enhance primary productivity for Sangiovese (Bindi *et al.* 2001), Cabernet Sauvignon, and Riesling (Wohlfahrt *et al.* 2018) in FACE experiments, as well as for Tempranillo tested under a temperature gradient greenhouse (Arrizabalaga-Arriazu *et al.* 2021). Yet, there is a greater need to understand how CO_2_ concentration and water deficit interact to influence the allocation of biomass to roots versus shoots in different scion–rootstock combinations of grapevines to guide adaptations.

### Evapotranspiration and stomatal conductance reveal varied water use strategies

We found that integrating total leaf area with a gravimetric approach significantly improves estimates of water loss and complemented stomatal conductance data. Specifically, our regression analysis shows that Chardonnay had a larger range in conductance values and a higher conductance maximum associated with a greater evapotranspiration maximum than Cabernet Sauvignon (Fig. 5). We standardized water loss based on evapotranspiration per unit leaf area finding that for vines grown under ambient CO_2_ conditions, well-watered Chardonnay used 43% more water than Cabernet Sauvignon. This complements our stomatal conductance results showing higher conductance values for Chardonnay under the same conditions. Chardonnay also exhibited a greater decline in evapotranspiration rates when exposed to a water-deficit treatment in comparison to Cabernet Sauvignon (Fig. 4), which again was reflected in more conductance sensitivity observed for Chardonnay, particularly under ambient CO_2_. Lastly, evapotranspiration per unit leaf area was reduced under the 700 ppm treatment for Cabernet Sauvignon and Chardonnay demonstrating transpiration may be alleviated in a future CO_2_ environment (Fig. 4). This finding contrasts with a FACE study on Cabernet Sauvignon and Riesling, where transpiration rates derived from gas exchange measurements were higher under elevated CO_2_ for three consecutive growing seasons (Wohlfahrt *et al.* 2018); however, this previous study also suggested that potential interactions with soil water availability may have influenced their results. Experiments on other plants such as Cassava and Arabidopsis suggest decreases in transpiration under elevated CO_2_ environments (Teng *et al.* 2006; Cruz *et al.* 2018), which were linked to a reduction in conductance and stomatal density (Teng *et al.* 2006). Our results corroborate recent findings on other crops, such as wheat and sage, that elevated CO_2_ and water stress have genotype-dependent effects on leaf gas exchange (Medina *et al.* 2016; Javaid *et al.* 2022). However, we did not identify any other study that used a similar method coupling gravimetric water loss and estimated leaf area to infer differences in water use between two grapevine varieties.

### Genotypic variability may provide useful adaptation mechanisms

We demonstrate that Cabernet Sauvignon and Chardonnay, two varieties (genotypes) of the same species (*V. vinifera* L.), reveal nuances in their water use strategies as evidenced by their leaf level conductance and evapotranspiration per unit leaf area. However, they did not necessarily reflect two opposing hydric classifications as explained by the iso-anisohydric theory (Tardieu and Simonneau 1998). In this study, despite Chardonnay having higher conductance values and transpiration rates under ambient conditions (typical of anisohydric plants), it was also more sensitive to water deficit than Cabernet Sauvignon, lowering stomatal conductance to a greater degree (a behaviour observed in isohydric plants). Our findings align more with the interpretations of Hochberg *et al.* (2018) that this dichotomy is not an intrinsic property of the plant but instead are a function of plant–environment interaction, and that these opposing strategies alone do not dictate which mechanism is more favourable under water-deficit conditions (Martínez-Vilalta and Garcia-Forner 2017; Chen *et al.* 2019; Serrano *et al.* 2024), or in combination with other environmental stressors such as heat, light, and elevated CO_2_.

## Conclusions

In this controlled growth chamber study, we found that elevated CO_2_ promotes grapevine *i*WUE, particularly when exposed to short-term water deficit, and that this positive effect was greater for Cabernet Sauvignon than for Chardonnay. Contrary to our predictions, the increase in *i*WUE observed for both varieties were mediated by a reduction in stomatal conductance in response to elevated CO_2_ rather than an increase in photosynthesis. Instead, we found a decrease in photosynthesis for both varieties in response to elevated CO_2_, and this negative effect was greater for Chardonnay when well-watered. We explained this as a nitrogen dilution effect for Cabernet Sauvignon but not for Chardonnay. However, the underlying cause of downregulation under elevated CO_2_ environments is unclear and requires follow-up studies to examine the various mechanisms contributing to photosynthetic efficiency. Collectively, the conductance and evapotranspiration results indicate Cabernet Sauvignon is not only more water-conservative than Chardonnay, but also less sensitive to water deficit and elevated CO_2_. Based on these findings, we conclude Cabernet Sauvignon is the more sustainable variety to grow in water-limited climates and as atmospheric CO_2_ continues to rise. However, we encourage similar chamber studies to be reproduced with larger sample sizes to support the generalizability of these findings, particularly when making climate-adaptive management choices in viticulture.

## Supplementary Material

plaf011_suppl_Supplementary_Materials

## Data Availability

All data and code supporting these findings are incorporated in this article and in its online Supplementary Material.
